# Comparative analysis of robotic gastrectomy and laparoscopic gastrectomy for gastric cancer in terms of their long-term oncological outcomes: a meta-analysis of 3410 gastric cancer patients

**DOI:** 10.1186/s12957-019-1628-2

**Published:** 2019-05-23

**Authors:** Guixiang Liao, Zhihong Zhao, Muhammad Khan, Yawei Yuan, Xianming Li

**Affiliations:** 1 Department of Radiation Oncology, Shenzhen People’s Hospital, Second Clinical Medicine Centre, Jinan University, Shenzhen, 518020 People’s Republic of China; 2 Department of Nephrology, Shenzhen People’s Hospital, Second Clinical Medicine Centre, Jinan University, Shenzhen, People’s Republic of China; 3 Department of Oncology, First Affiliated Hospital of Anhui Medical University, Hefei, People’s Republic of China; 4 Department of Radiation Oncology, Cancer Center of Guangzhou Medical University, Guangzhou, Guangdong People’s Republic of China

**Keywords:** Robotic gastrectomy, Laparoscopic surgery, Gastric cancer, Overall survival (OS), Prognosis

## Abstract

**Background:**

Data regarding the long-term oncological outcomes of robotic gastrectomy (RG) are limited despite the increased commonality of this method as an alternative for gastric cancer treatment. Here, we conducted a meta-analysis to evaluate the long-term oncological outcomes of RG in comparison to that of laparoscopic gastrectomy (LG).

**Methods:**

The PubMed, ISI Web of Science, EMBASE, and Cochrane Library databases were comprehensively searched for studies that compared RG and LG in terms of their long-term survival outcomes. The hazard ratios (HRs) of overall survival (OS), disease-free survival (DFS), and relapse-free survival (RFS) were obtained, while the odds ratio (OR) was recorded for the recurrence rate. A sensitivity analysis was performed. Egger’s test and Begg’s test were applied to evaluate publication bias.

**Results:**

Eight studies were identified and involved 3410 gastric cancer patients (RG, 1009; LG, 2401). The two groups had no significant differences in OS (HR, 0.98; 95% CI, 0.80–1.20; *P* = 0.81), DFS (HR, 1.36; 95% CI, 0.33–5.59; *P* = 0.67), RFS (HR, 0.92; 95% CI, 0.72–1.19; *P* = 0.53), or recurrence rate (OR, 0.92; 95% CI, 0.71–1.19; *P* = 0.53). Moreover, the two techniques were comparable in length of hospital stay (LOS), postoperative complication rate, 30-day mortality rate, and rate of conversion to open surgery.

**Conclusions:**

The long-term oncological outcomes, expressed as OS, DFS, RFS, and recurrence rate, were similar between RG and LG. However, more randomized controlled trials with rigorous study designs and patient cohorts are needed to evaluate the oncologic outcomes of RG in patients with gastric cancer.

**Electronic supplementary material:**

The online version of this article (10.1186/s12957-019-1628-2) contains supplementary material, which is available to authorized users.

## Introduction

Gastric cancer (GC) is the third leading cause of cancer death, with 782,685 (8.2%) deaths (both sexes, all ages) and 1,033,701 (5.7%) new cases (both sexes, all ages) in 2018, making GC the fifth most common cancer worldwide [1]. Despite progress in multidisciplinary therapy, radical excision is regarded as the most effective curative treatment approach [2]. The safety and feasibility of laparoscopic gastrectomy (LG) are confirmed in the literature [3–5]. However, conventional laparoscopy has some limitations, including poor imaging, loss of faculties, and a long learning curve [6]. The application of robotic-assisted devices may surmount the technical drawbacks of laparoscopic surgery.

Many studies have demonstrated the safety and feasibility of treating gastric cancer with RG regarding the intraoperative outcomes and short-term outcomes. Some studies indicate that RG and LG are comparable [7–9]. However, the data are limited and mostly focus on the prognosis of RG; thus, we conducted an updated meta-analysis to provide a more comprehensive assessment of the two techniques.

## Methods

### Search strategies

The Preferred Reporting Items for Systematic Reviews and Meta-Analyses statement was applied to perform the meta-analysis [10]. The search was independently performed by two authors (G.L. and Z.Z.) in October 3, 2018. The databases included PubMed (from 1980 to October 2018), ISI Web of Science (from 2000 to October 2018), Cochrane Library (from 1950 to October 2018), and EMBASE (from January 1990 to October 2018). The following search terms were employed: robotic, gastric cancer, gastrectomy, survival, and prognosis. The search strategy is shown in Additional file 1. The search had no language restrictions.

### Inclusion and exclusion criteria

The search was independently conducted by two reviewers (G.L. and Z.Z.). The relevant data were recorded by the reviewers (G.L. and Z.Z.). If there was a disagreement, the reviewers discussed the issue and finally made a decision.

The inclusion criteria were as follows: (1) studies focused on resectable gastric cancer, (2) studies comparing RG and LG to evaluate the safety and feasibility of each technique, and (3) studies that effectively reported the prognosis after surgical intervention, and the prognosis was described in terms of overall survival (OS), disease-free survival (DFS), recurrence-free survival (RFS), or the recurrence rate. The time from surgery to all-cause death or the last follow-up in the study was defined as OS. The time to cancer recurrence, development of a second cancer, or disease development was defined as DFS. The time from surgery to cancer recurrence was defined as RFS.

#### Exclusion criteria

The exclusion criteria were as follows: (1) studies that were not focused on resectable gastric cancer; (2) studies that were non-comparative studies; (3) studies that were reviews or conference abstracts and were not human studies.

### Outcomes of interest and data extraction

Two authors (G.L. and Z.Z.) carefully and independently reviewed the included studies and extracted the effective data in a standard form. The data included the basic characteristics of the study (author, publication year, country, cases in each group, age, body mass index, TNM stage, and the number of harvested lymph nodes). The primary outcomes were the survival outcomes, including OS, DFS, RFS and recurrence rate. The secondary outcomes included the length of hospital stay (LOS), postoperative complications, 30-day mortality, and the rate of conversion to open surgery. The follow-up time of each included study was recorded. If different opinions existed, the authors conducted a full discussion and a final decision was made.

### Quality assessment

The quality of the included studies was evaluated with the Newcastle-Ottawa Scale. This quality assessment evaluated the cohort selection, comparability, and ascertainment of the outcomes [11]. The study quality was divided into three categories: poor, fair, and good.

### Statistical analysis

Review Manager 5.3 software (Collaboration, Oxford, UK) was applied for pooling the meta-analysis. If the survival data were provided with HRs and 95% CIs, we extracted the data directly for meta-analysis. Otherwise, if the survival data were shown as Kaplan-Meier survival curves, we used the Engauge Digitizer (version 4.1) to estimate the HRs and 95% CIs and reconstruct the HR and standard error (SE), as described by Tierney et al. [12]. Dichotomous parameters were recorded as odds ratios (ORs) and 95% CIs. Risk difference (RD) was applied if both groups had no event occurrences. Continuous variables were assessed using the mean difference (MD). The *I*^*2*^ statistic was applied to test heterogeneity. If *I*^*2*^ < 50%, a fixed-effects model was adopted. Otherwise, the random-effects model was adopted. Sensitivity analysis was performed. Egger’s test and Begg’s test were applied to assess publication bias. The significance level was set at *P* < 0.05.

## Results

**Fig. 1:**
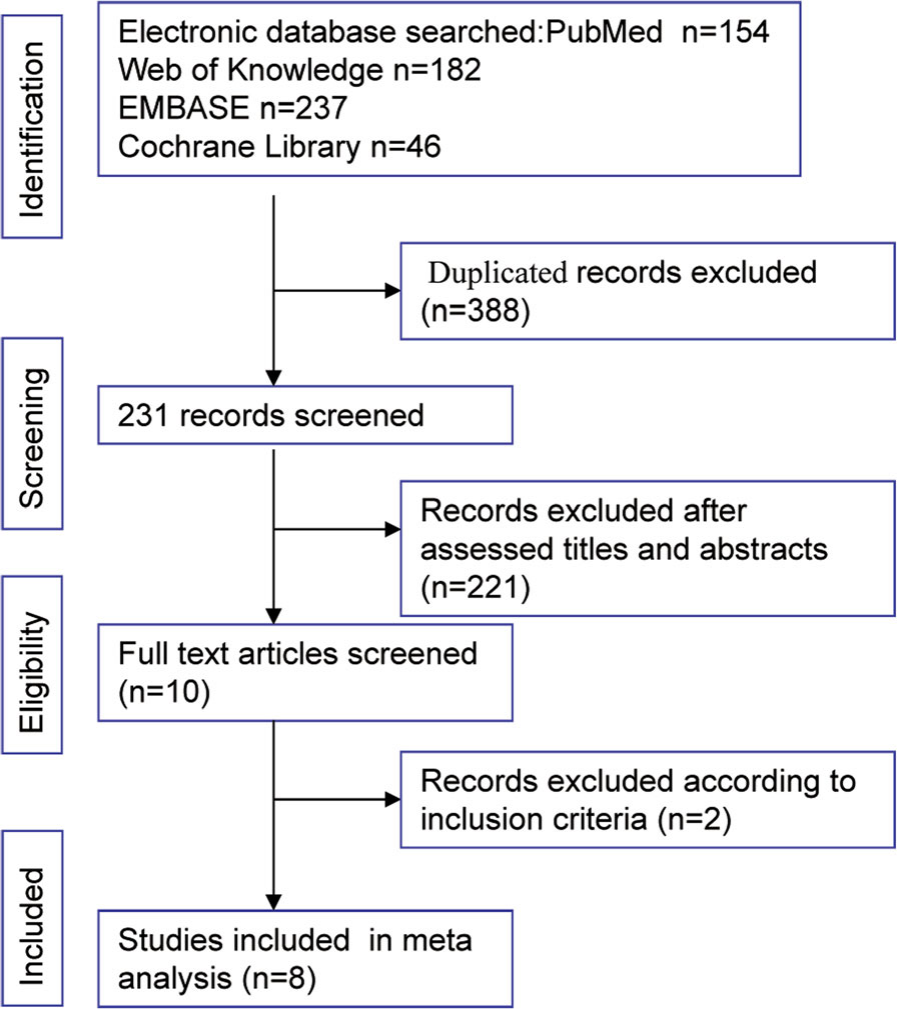
The study selection process

Our comprehensive literature search identified 589 studies. There were 154 papers from PubMed, 182 papers from Web of Knowledge electronic database, 237 papers from EMBASE, and 46 papers from the Cochrane Library. We used Endnote software and removed 388 duplicated papers. The titles and abstracts of the remaining 231 papers were screened, and 10 papers potentially suitable for analysis were identified. After a thorough review of the full text of the 10 papers, one paper was excluded because it was a letter [13], and another study was excluded because the survival data were insufficient for analysis [14]. Finally, eight papers were included for our meta-analysis [15–22]. The study selection process is shown in Fig. 1. The basic characteristics are presented in Table 1. There were three papers from China, two papers from Japan, two papers from Korea, and one paper from Italy. Three studies were published in 2018 and included propensity score matching (PSM) analyses. In total, 1009 (29.60%) gastric cancer patients underwent RG, and 2401 (70.41%) gastric cancer patients underwent LG. All of the included studies were retrospective studies. The quality was fair for the study from Pugliese et al. [21] and was good in the rest of the studies.

**Table 1:** The general characteristics of the extracted data and quality of the included studies

Study	Country	Study design	Group	Cases (*n*)	Age (mean (SD) or median)	BMI (mean (SD) or median)	TNM stage (I/II/II/IV)	OS (3-year or 5-year)	RFS (3-year or 5-year)	Relapse (*n*)	HLN ((mean (SD) or median)	Follow-up time (median (range) (M)	Quality
Gao Y 2018	China	R	RG LG	163 339	60.27 ± 10.50 59.36 ± 11.08	23.77 ± 3.11 23.44 ± 3.47	0/57/106/0 0/169/170/0	76.1% 81.7%	73.0% 67.6%	44 106	30.55 ± 10.13 29.34 ± 9.76	50.5 (36–72)	good
Lee J 2015	Korea	R	RG LG	133 267	53.6 ± 13.2 59.2 ± 11.7	23.2 ± 2.7 23.7 ± 2.8	101/15/17/0 218/32/17/0	94.7% 93.2%	NA	NA	41.2 ± 13.1 39.9 ± 13.3	75 (36–126)	good
Li Z 2018	China	R	RG LG	125 329	55.4 ± 11.5 56.9 ± 10.5	23.7 ± 2.8 23.1 ± 3.0	24/51/50/0 37/127/165/0	78.6% 74.1%	81.2% 78.6%	21 24	29.5 ± 9.6 27.7 ± 8.7	28 (3–52)	good
Nakauchi M 2016	Japan	R	RG LG	84 437	64 68	22.6 21.8	61/10/12/1 310/80/42/5	86.9% 88.8%	86.9% 86.3%	11 60	40 38	42.2 (1.7–78.9)	good
Obama K 2017	Japan	R	RG LG	315 525	54.5 ± 12.6 59.3 ± 11.9	23.6 ± 3.1 23.5 ± 2.9	254/30/31/0 441/64/20/0	93.3% 91.6%	90.7% 90.5%	21 26	40.1 ± 15.4 38.6 ± 14.5	85 (60–114)	good
Pugliese R 2010	Italy	R	RG LG	18 52	NA	NA	All (50/8/9/3)	78% 85%	NA	4 8	25 ± 4.5 31 ± 8	53 (3–112)	fair
Son T 2014	Korea	R	RG LG	51 58	55.3 ± 12.2 58.8 ± 12.2	22.7 ± 2.9 23.2 ± 3.3	35/8/8/0 43/10/5/0	89.5% 91.1%	90.2%^a^ 91.2%^a^	3 3	47.2, 42.8	70 (24–112)	good
Zhou J 2014	China	R	RG LG	120 394	54.7 55.6	21.6 ± 2.8 21.7 ± 2.6	29/36/55/0 115/98/181/0	67.8% 69.9%	NA	5 28	34.6 ± 10.9 32.7 ± 11.2	17 (3–41)	good

*BMI* body mass index, *HLN* harvested lymph nodes, *M* months, *NA not available, OS* overall survival, *R* retrospective, *RFS* relapse-free survival, *SD* standard deviation. ^a^Disease-free survival

### The primary outcomes

**Fig. 2:**
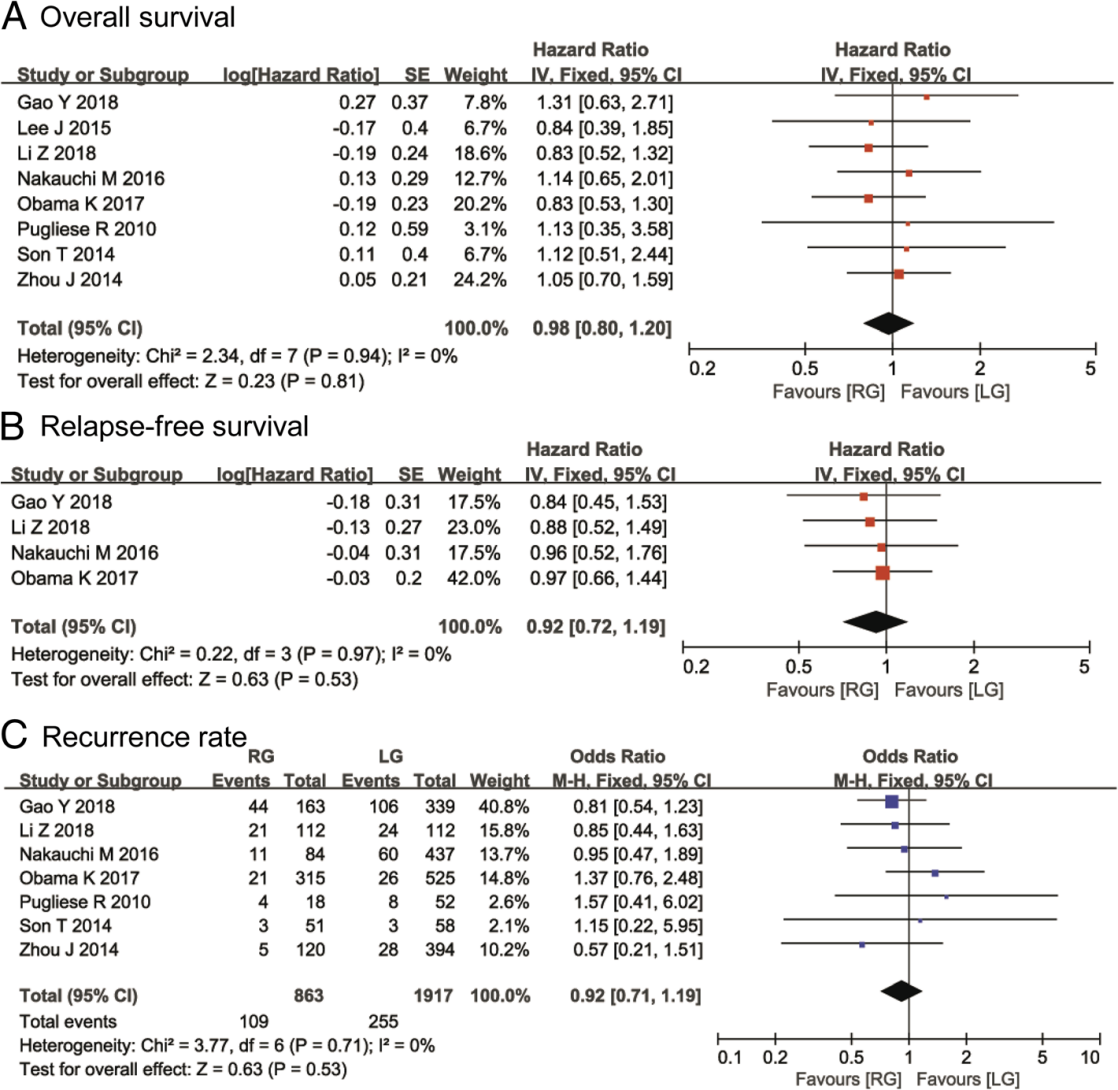
Meta-analysis of survival outcomes between robotic gastrectomy (RG) and laparoscopic gastrectomy (LG): **a** overall survival, **b** relapse-free survival, and **c** recurrence rate

All of the included studies reported OS outcomes [15–22]. The pooled data from the eight studies indicated no significant difference between the two groups (HR, 0.98; 95% CI, 0.80–1.20; *P* = 0.81), and a fixed-effects model was adopted due to the lack of significant heterogeneity *(I*^*2*^ = 0, *P* = 0.94, Fig. 2a). Only one study reported DFS results [16]; there was no significant difference between the two groups in DFS, the HR was 1.08 with a 95% CI from 0.26 to 4.44, and the *P* value was 0.67. Four studies described the RFS results [15–17, 20]. A meta-analysis of the four studies indicated that the two techniques had similar RFS outcomes (HR, 0.92; 95% CI, 0.72–1.19; *P* = 0.53). The analysis showed no significant heterogeneity (*I*^*2*^ = 0%, *P* = 0.97) using a fixed-effects model (Fig. 2b). Seven studies, with a total of 2780 gastric cancer patients, reported recurrence rates. In total, the recurrence rate was 12.63% (109/863) in the RG group and 13.30% (255/1917) in the LG group. The pooled data from the seven studies suggested that the recurrence rate was similar between the two techniques (OR, 0.92; 95% CI, 0.71–1.19; *P* = 0.53). The analysis had no obvious heterogeneity (*I*^*2*^ = 0, *P* = 0.71) (Fig. 2c).

### The secondary outcomes

**Fig. 3:**
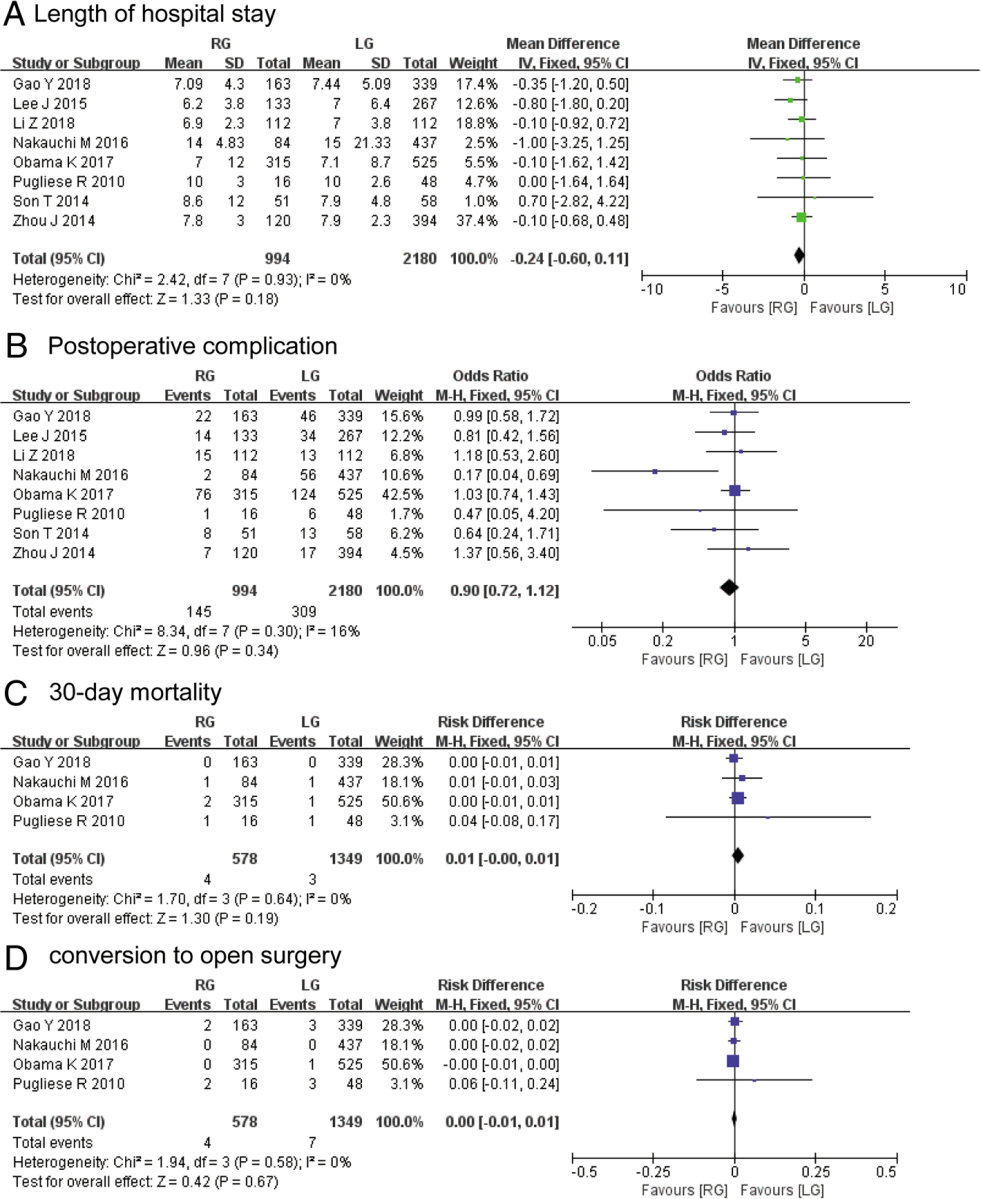
Pooled data for the outcomes of interest between patients who underwent robotic gastrectomy (RG) and laparoscopic gastrectomy (LG): **a** length of hospital stay, **b** postoperative complication, **c** 30-day mortality, and **d** conversion to open surgery

The two techniques had a comparable LOS (MD, − 0.24; 95% CI, − 0.60 to 0.11; *P* = 0.16) based on the pooled data from all the included studies [12, 15–22], and a fixed- effect model was adopted because there was no heterogeneity (Fig. 3a). Moreover, the two groups had similar rates in postoperative complications (OR, 0.90; 95% CI, 0.72–1.12; *P* = 0.34) (Fig. 3b), 30-day mortality (RD, 0.01; 95% CI, 0.00–0.01; *P* = 0.19) (Fig. 3c), and conversion to open surgery (RD, 0.00; 95% CI, − 0.01 to 0.01, *P* = 0.67) (Fig. 3d). These results were all measured using fixed- effects models due to the lack of significant heterogeneity.

### Sensitivity analysis

**Fig. 4:**
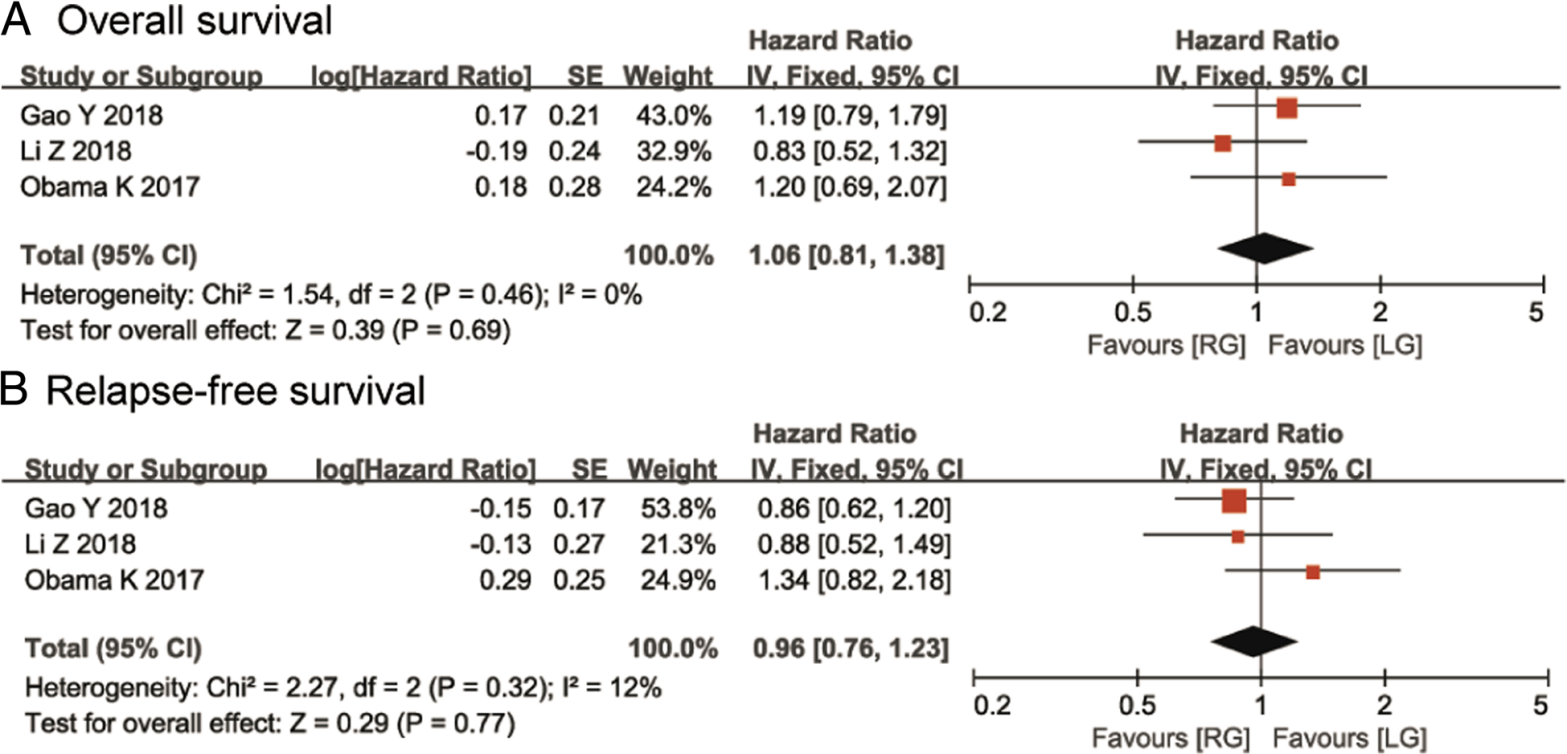
Sensitivity analysis of the studies that performed propensity score matching analyses for **a** overall survival and for **b** relapse-free survival

Three papers performed PSM analyses. We conducted a sensitivity analysis for papers that performed PSM analyses [15–17]. In terms of OS, the results showed that there was no significant difference between the two techniques (HR, 1.06; 95% CI, 0.81–1.38; *P* = 0.69) (Fig. 4a). In terms of RFS, the result was not influenced by the surgical technique (HR, 0.96; 95% CI, 0.76–1.23; *P* = 0.77), and there was no obvious heterogeneity (*I*^*2*^ = 12, *P* = 0.32) (Fig. 4b).

### Publication bias

**Fig. 5:**
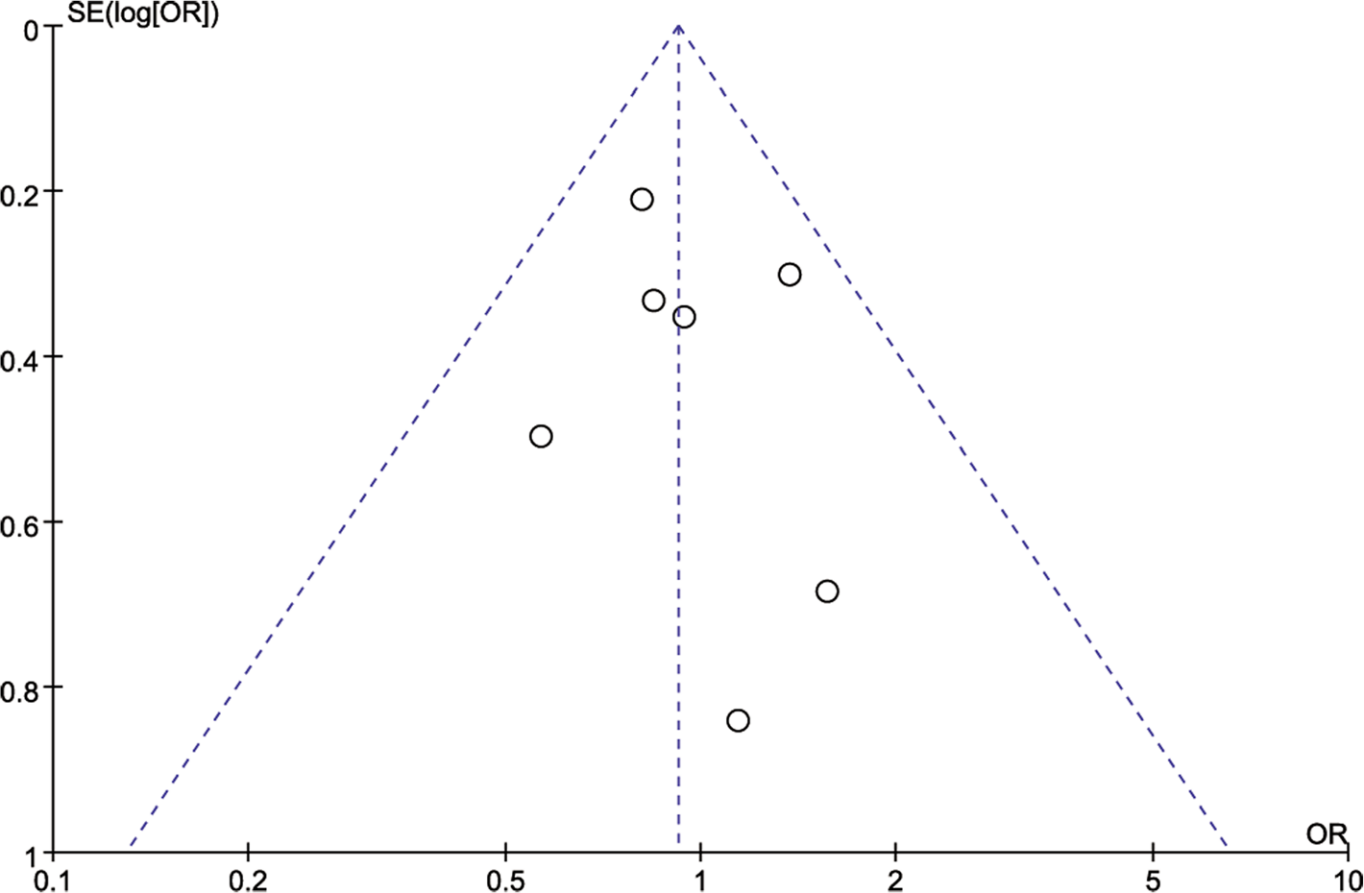
Funnel plot of the relapse rate between patients who underwent robotic gastrectomy (RG) and laparoscopic gastrectomy (LG). OR, odds ratio; SE, standard error

A funnel plot for recurrence rate was adopted to assess publication bias. No evidence of publication bias was found, and all the results were within the 95% CI (Fig. 5). The statistical analysis revealed no significant publication bias (Begg’s test *P* = 0.764 and Egger’s test *P* = 0.571).

## Discussion

Currently, the benefits of treating gastric cancer with the laparoscopic technique have been well recognized. Many documents have indicated that compared to open surgery, laparoscopic surgery can improve the safety and efficacy of treating gastric cancer [23–26]. However, there are some limitations to laparoscopic gastric surgery. These drawbacks include poor imaging, inconvenient lymph node dissection in a narrow space, technical complexity, and a long and steep learning curve [27]. In an effort to address the drawbacks of the laparoscopic technique, robotic-assisted surgical systems were introduced. These surgical systems could offer some improvements in visibility and manipulation [28].

A body of literature has been published and indicates that RG is as safe and effective as LG [9, 29, 30]. Moreover, RG could reduce intraoperative blood loss and reduce the length of the hospital stay but requires a longer operative time [31].

In contrast, only a few studies focus on prognosis after RG as this technology has only existed for less than 20 years. However, as GC is a malignant tumor, the prognosis and long-term outcomes of GC patients are major concerns for surgeons [32]. Recently, some studies focusing on the prognosis after RG and LG have been published [15–17]. Hence, we performed this study to evaluate the long-term outcomes associated with the two techniques.

Our meta-analysis included eight studies involving 3410 patients. The results of our meta-analysis revealed no significant differences in OS, DFS, RFS, and recurrence rate between the RG and LG groups. These results indicate that the two approaches have similar long-term oncologic outcomes. Moreover, the two techniques were comparable in LOS, postoperative complication rate, 30-day mortality rate, and rate of conversion to open surgery. In the sensitivity analysis, the OS and RFS outcomes were not influenced after PSM between the two approaches. These results indicate that for the management of gastric cancer, RG is a safe technique in terms of oncologic outcomes.

The treatment of gastric cancer requires a multidisciplinary effort. It is recommended to include surgery, chemotherapy, and radiation therapy in treating gastric cancer [33]. For gastric cancer surgery, achieving R0 resection is a vital factor for prognosis, while R1 and R2 resection might predict worse survival outcomes [34, 35]. The benefits of RG in terms of visibility and manipulation might provide a good quality of surgery and thus might produce good oncologic outcomes. OS is a major oncologic outcome. In this study, the five-year OS was similar to that previously reported [36]. Furthermore, the results also indicate the oncological safety of RG as well as that of LG.

It is reported that the recurrence rate in gastric cancer patients is nearly 50% within 5 years after surgery [37], and 50–90% of patients die of tumour relapse [38]. To the best of our knowledge, no meta-analysis has previously reported RFS with RG and LG. Our meta-analysis indicates no significant difference in the two groups in terms of the RFS outcome. Moreover, in this meta-analysis, seven studies reported recurrence rates. The RFS and recurrence rate results further prove the comparability between RG and LG in terms of long-term oncological outcomes. It has been reported that after LG, lymph node metastasis can predict the recurrence of gastric cancer [39]. Nakauchi et al. demonstrated similar profiles for sites of recurrence between the two groups [20]. The recurrence rate was 12.63% in the RG group and 13.30% in the LG group. The recurrence rate in the RG group was similar to that in the LG group.

Moreover, no significant difference was found in the LOS between the two approaches. This was consistent with a previous meta-analysis [40]. Regarding the postoperative complication rate and 30-day mortality rate, the rates were equivalent between the two groups [6–8]. Furthermore, the comparable rate of conversion to open surgery also indicated that RG was as safe and effective as LG [6–8].

To minimize selection bias in these two approaches, we performed a sensitivity meta-analysis based on the studies that applied PSM. The results also indicate that the two groups had no significant differences.

Limitations in this meta-analysis should be taken into account. First, the studies included for analysis were retrospective studies, and none of the studies were randomized controlled trials. Pooling the retrospective studies may affect the effective power of an intervention [4], which could result in publication bias. However, there was no significant publication bias in this meta-analysis. Second, some studies did not provide HRs and SEs directly, and these data were extracted from the survival curves, which could introduce a potential source of bias. Third, adjuvant treatment might have been applied for the patients, and the differences in adjuvant treatment might also affect the individual survival time. Fourth, seven out of the eight included studies were located in East Asian countries, and the data regarding Western countries are limited. The applicability and generalizability of these results are limited. These results should be interpreted with caution. Fifth, most studies were from a single centre in Eastern countries. Multi-centre, prospective randomized controlled trials and high-quality studies are needed in the future. Fortunately, a randomized controlled trial study is ongoing [41].

## Conclusion

RG is safe and effective and is comparable to LG. The two groups had similar OS, DFS, RFS, and recurrence rates after a long-term follow-up. Moreover, more randomized controlled trials with rigorous study designs and patient cohorts are needed to evaluate the long-term outcomes of RG in patients with gastric cancer.

## Additional file

Additional file 1: Search strategies. (DOCX 15 kb)

## Data Availability

The data in this manuscript are all provided in the tables and texts.
