# Supplementary material for: Comparative analysis of robotic gastrectomy and laparoscopic gastrectomy for gastric cancer in terms of their long-term oncological outcomes: a meta-analysis of 3410 gastric cancer patients
Source: World J Surg Oncol. 2019 May 23;17:86. doi: 10.1186/s12957-019-1628-2 (PMC6533666; doi:10.1186/s12957-019-1628-2)
Supplement: Supplementary file 1 — Search strategies. (DOCX 15 kb) [file 12957_2019_1628_MOESM1_ESM.docx]

Pubmed

#1 (gastric or stomach or gastri*)[All Fieids]

#2 (cancer or tumor or carcinoma or tumour or neoplasm or malignant or lesion)[All Fieids]

#3 (da vinci surgical system or Zeus or computer assist* surger* or robot* or robotic assist* surger*)[All Fieids]

#4 (gastrectomy or gastrec* or surgery)[All Fieids]

#5 (prognosis or predict* or survival or survi* or prognos*)[All Fieids]

#6 (#1 AND #2 AND #3 AND #4 AND #5)

Web of Science

#1 TS=(gastric or stomach or gastri*)

#2 TS=(cancer or tumor or carcinoma or tumour or neoplasm or malignant or lesion)

#3 TS=(da vinci surgical system or Zeus or computer assist* surger* or robot* or robotic assist* surger*)

#4 TS=(gastrectomy or gastrec* or surgery)

#5 TS=(prognosis or predict* or survival or survi* or prognos*)

#6 (#1 AND #2 AND #3 AND #4 AND #5)

Cochrance library

#1 gastric OR stomach[title abstract keyword]

#2(cancer or carcinoma or tumo* or neoplas* or tumour or adenocarcinom* or adenom* or lesion* or malign*)[title abstract keyword]

#3( da vinci surgical system or Zeus or computer assist* surger* or robot* or robotic assist* surger*)[title abstract keyword]

#4(gastrectomy or gastrec* or surgery) [title abstract keyword]

#5 (prognosis or predict* or survival or surv* or prognos*)[title abstract keyword]

#6( #1 AND #2 AND #3 AND #4 AND #5)

EMBASE

#1 exp gastric cancer

#2 exp gastric tumor

#3 exp gastric carcinoma

#4((gastric or stomach) adj3 (cancer or carcinoma or tumo* or neoplas* or tumour or adenocarcinom* or adenom* or lesion* or malign*)).mp

#5(#1 OR #2 OR #3 OR #4)

#6 exp robotics

#7 (da vinci surgical system or Zeus or computer assist* surger* or robot* or robotic assist* surger*).mp

#8(#6 OR #7)

#9 exp gastric surgery,gastrectomy

#10(gastric surgery or gastrectomy).mp

#11(#9 AND #10)

#12(#5 AND #8 AND#11)

#13 exp prognosis

#14 exp predict

#15 exp survival

#16 (survi* or predict* or prognost*).mp

#17(#13 OR #14 OR #15 OR #16)

#18(#12 AND #17)
